# Serologic Rebound after Stopping Azoles for Primary Pulmonary Coccidioidomycosis: A Case-Controlled Observational Study

**DOI:** 10.3390/jof9090901

**Published:** 2023-09-01

**Authors:** Priyal J. Shah, Neil M. Ampel, Marlene E. Girardo, Janis E. Blair

**Affiliations:** 1 Division of Infectious Diseases, Mayo Clinic, Phoenix, AZ 85054, USA; priyaljshah@gmail.com (P.J.S.); ampel.neil@mayo.edu (N.M.A.); 2 Department of Quantitative Health Sciences, Mayo Clinic, Scottsdale, AZ 85059, USA; girardo.marlene@mayo.edu

**Keywords:** coccidioidomycosis, complement fixation, extra-thoracic dissemination, fluconazole, primary pulmonary coccidioidomycosis, Valley fever, serology

## Abstract

Background: We sought to characterize the outcomes of patients with primary pulmonary coccidioidomycosis whose post-treatment complement fixation (CF) titer increased by more than 2 dilutions (serologic rebound) after discontinuation of antifungal treatment. Methods. We conducted a retrospective chart review of patients with primary pulmonary coccidioidomycosis and identified immunocompetent, non-pregnant adults who received antifungal treatment and then experienced a serologic rebound after treatment discontinuation. We compared these to matched controls similarly treated who did not have serologic rebound. Results. Fifty-eight patients experienced serologic rebound. Thirty (52%) of these were associated with symptoms. Nine were associated with radiographic progression. The median time to serologic rebound was 3.5 months. Antifungal treatment was reinitiated in 37 (63.7%) patients. Four of the 58 (6.9%) with rebounded serology subsequently developed extra-thoracic dissemination. Compared with matched controls, patients with rebounded serology were more likely to have post-treatment symptoms, reinitiation of antifungal therapy, and a longer duration of clinical follow-up. However, they were not more likely to experience extra-thoracic dissemination. Conclusion: Serological rebound, manifested in at least 2-dilution rise of CF titer following antifungal treatment of primary pulmonary coccidioidomycosis, was uncommon, but resulted in longer clinical follow-up. Continued monitoring of such patients is important to identify the patients who develop subsequent symptoms, as well as extra-thoracic dissemination.

## 1. Introduction

Pulmonary coccidioidomycosis is a common fungal infection of the desert southwestern United States, as well as northern Mexico and areas of Central and South America. Roughly two thirds of cases of primary coccidioidomycosis are asymptomatic or minimally symptomatic and do not require medical attention or antifungal therapy [1,2]. For otherwise healthy patients who are immunocompetent, antifungal therapy is often reserved for those with moderate-to-severe pulmonary infection or for those at risk for developing severe disease [3]. An important tool in monitoring patients for progress in convalescence of their infection is the quantitative complement fixation (CF) serology [4,5]. Following infection, the complement fixation titer is detected (within a few-to-several weeks), slowly rises with time (over the course of months), then begins to fall concurrent with a patient’s clinical recovery, and eventually becomes negative with resolution of infection. The CF titer has an inverse relationship to the control of infection, and higher titers frequently correlate with more severe disease and the development of extra-thoracic dissemination [5]. Such concepts were described decades ago and originated from a single research laboratory, and have not been systematically corroborated despite many changes in how these tests have evolved since that time.

We have observed that following completion of antifungal therapy, complement fixation titers that initially decline during treatment may subsequently rebound, even when the patient is clinically stable and has no symptoms. Because such a rebound of complement fixation titers could portend a clinical relapse of infection or development of dissemination, we conducted this study to summarize our experience in such situations.

## 2. Material and Methods

This study was approved by the Mayo Clinic Institutional Review Board, and the requirement for consent was waived by the Institutional Review Board for this minimal-risk study. We searched the Mayo Clinic Arizona electronic health records from 1 January 2000, through 31 December 2020, using the International Classifications of Diseases nineth revision (ICD-9) and tenth revision (ICD-10) codes for acute pulmonary coccidioidomycosis and primary pulmonary coccidioidomycosis (ICD-9 114.0 and 114.5) and (ICD-10 B38.0 and B38.2), respectively. Cases included adult patients with ages greater than or equal to 18 years, diagnosed with proven or probable primary pulmonary coccidioidomycosis, who received a course of antifungal treatment, and whose complement fixation titer increased by 2 or more dilutions after discontinuation of the primary course of antifungal treatment. We excluded patients who were immunosuppressed, pregnant, or presented with chronic pulmonary or extrapulmonary infection. Matched controls were identified by an electronic search of immunocompetent patients with primary pulmonary coccidioidomycosis who were treated with a course of antifungal treatment, matched for age, sex, and race. When we were unable to identify an age-, sex-, and race-matched control, we prioritized race and age.

We defined proven and probable primary pulmonary coccidioidomycosis in accordance with the revised European Organization for Research and Treatment of Cancer and the Mycoses Study Group Education and Research Consortium [6]. Briefly, proven infection required a specimen with a positive culture or polymerase chain reaction for *Coccidioides* or a specimen with spherules identified on pathology or cytology. Probable infection required that a patient have compatible symptoms, accompanied by typical radiographic findings and positive coccidioidal serology. We define positive serology as any positive immunoglobulin G (IgG) by enzyme immunoassay, immunodiffusion or complement fixation, or immunoglobulin M (IgM) by immunodiffusion. Complement fixation studies were performed at the Mayo Clinic Infectious Diseases Serology Laboratory in Rochester Minnesota according to the previously described Laboratory Branch complement fixation test of the Centers for Disease Control and Prevention [5].

We defined serologic rebound when the patient experienced a rise in complement fixation titer of at least two dilutions above the titer measured at the time of antifungal discontinuation. We defined “asymptomatic rebound” when patients had no symptoms (other than fatigue) accompanying their increased serologic titer, and “relapse” when the patient experienced recurrent coccidioidal symptoms other than fatigue at the time of serologic rebound, accompanied by progressive radiographic imaging. We further defined “resolved infection” when the patient was asymptomatic and off antifungal treatment, had resolved their complement fixation, and had resolution of initial radiographic abnormalities.

We defined immunocompetent individuals as those who did not receive any immunosuppressing medications (such as, but not limited to, systemic corticosteroids, anti-rejection treatments for organ transplant recipients, biologic agents or disease-modifying antirheumatic agents for those with autoimmune or inflammatory conditions, cancer treatments) or whose illnesses rendered them innately immunosuppressed (such as, but not limited to, those with hematologic malignancies, human immunodeficiency virus infection with a CD4 count of less than 200 cells per microliter, or known innate immunodeficiencies).

We abstracted the following information from the medical record: demographic characteristics, past or concurrent medical conditions, information regarding coccidioidal illness (including symptoms present at initial presentation, symptoms present at the end of initial treatment, and symptoms present at the time of rebounded serology), all serial coccidioidal serologic results, radiographic findings over the time course of coccidioidal illness, and coccidioidal-specific antifungal treatments including medication, dose, and duration of treatment. We collected information for two years following the discontinuation of the initial antifungal treatment.

The primary outcome of this study was the presence or absence of extra-thoracic dissemination after treatment discontinuation. Secondary outcomes included the presence of relapsed infection, whether the patient required re-initiation of antifungal treatment, total duration of medical care in the coccidioidomycosis clinic, and the final status of the infection (i.e., resolved infection or requiring continued clinical observation) at the end of the two-year follow-up period.

Statistical analysis. We used descriptive statistics to summarize demographic and clinical characteristics for case and controls. We compared proportions of discrete variables and continuous variables using the Fisher exact test and *t*-test, respectively. We considered *p*-values of <0.05 to be significant. Statistical analysis was completed using R version 4.3.2.

## 3. Results

From 1 January 2000 through 31 December 2020, we identified 2969 patients with ICD codes for primary pulmonary coccidioidomycosis. From these 2969, we excluded patients for the following reasons: 1510 with inadequate records (including, but not limited to, the lack of follow-up serologies after stopping antifungal treatment); 168 with disseminated coccidioidomycosis at presentation; 133 did not receive antifungal treatment; 268 had positive serology according to immunodiffusion and/or enzyme immunoassay, but the complement fixation was negative; 46 had immunosuppressive medications or conditions; 191 had miscellaneous exclusions such as recognition of disseminated infection during the initial treatment course, received lifelong antifungal treatment, were pregnant at initial illness, or were younger than 18 years of age at coccidioidal diagnosis. After applying these exclusion criteria, we had a group of 653 immunocompetent non-pregnant patients with primary pulmonary coccidioidomycosis who received a discreet course of antifungal treatment and had available follow-up serologies. From these 653, 56 (8.6%) experienced serologic rebound (at least 2-dilution elevation) after stopping the antifungal treatment, and 597 did not have serologic rebound. Two additional patients meeting case definition were identified while searching for control subjects and included in the analysis of the case subjects.

Among these 58 patients with serologic rebound, 57 began treatment with fluconazole, and one with voriconazole. Two of the 57 switched to either voriconazole or itraconazole due to fluconazole intolerance. The median complement fixation titer at the time of diagnosis was 1:8 (range, 1:2–1:256), 1:2 (range, negative—1:32) at the end of antifungal therapy, and 1:16 (range 1:4–1:256) at the time of serologic rebound. The median time to serologic rebound was 3.5 months (range 0–21). Of these 58 patients, antifungal therapy was restarted in 37 (63.8%), including 23 of 30 (76.7%) persons who had symptoms associated with the serologic rebound, and 14 of 28 (50%) patients who remained asymptomatic. When we compared the demographics and outcomes of those with symptomatic relapse versus those with asymptomatic serologic rebound, those with symptoms were more likely to be white, but no differences were observed (Supplement Table S1). Among the 23 with symptomatic serologic rebound, symptoms at the time of rebound included: cough (11), fever (7), night sweats (5), dyspnea and chest pain (4 each), and headaches or body pain (3 each).

Nine (15.5%) of the 58 patients with serologic rebound had also had progression of radiographic abnormalities at the time of rebound, likely reflecting true relapsed infection, Seven of the nine were symptomatic; all nine received a second course of antifungal treatment, and eight recovered without further incident; one was lost to follow-up.

Four (6.9%) of the fifty-eight patients subsequently developed extra-thoracic coccidioidomycosis. All were white males aged 64–87 years. In three cases, the patients developed headache, nausea, and fever within 5 months of antifungal discontinuation and were found to have coccidioidal meningitis. In retrospect, at the time of their primary pulmonary coccidioidomycosis, none of these three patients had initial presentations characterized by marked headaches or other symptoms suggesting meningitis. The other patient developed bone dissemination 4 years after asymptomatic serological rebound for which he received an additional five months of antifungal treatment at the time of rebound. We did not find any differences in the demographic characteristics of those who experienced extra-thoracic dissemination and those who did not (Supplementary Table S2).

The patients with serologic rebound were compared to matched control subjects without serologic rebound (Table 1). Compared to controls, patients with serologic rebound were significantly more likely to develop symptoms after the initial course of antifungal therapy (52% vs. 22%; *p* < 0.001) and receive additional courses of antifungal therapy (64% vs. 9%; *p* < 0.001). There was one case of extra-thoracic dissemination in the control group, which was not statistically different from the four in the rebound group (6.9% vs. 1.7%, *p* = 0.40).

## 4. Discussion

Complement fixation serology was first developed for use in the diagnosis of coccidioidomycosis by Charles E. Smith in the 1940s [5,7], and he articulated many of the concepts that we hold today in the interpretation of serologic testing. He was the first to describe that complement fixation titers declined spontaneously over time as the patient recovered from primary coccidioidomycosis, and to caution when titers did not decline, because in general, a rising complement fixation titer was felt to reflect increasing fungal growth, portending worsening disease [5]. However, these concepts have not been independently corroborated, despite the changes in technique that have occurred over time.

Today, reference labs continue to conduct complement fixation testing for coccidioidomycosis, despite the fact that such testing is acknowledged to be a labor-intensive, manual process that requires interpretation by an experienced laboratorian. Additionally, because of the variabilities in individual laboratory processes and protocols, an individual should ideally have serial complement fixation testing conducted in the same laboratory over time [5].

In the current study, rebounding complement fixation titers that rose at least 2 dilutions after antifungal treatment discontinuation in primary pulmonary coccidioidomycosis was not an uncommon finding (56 of 653, 8.6%). Thirteen (22.4%) of the fifty-eight experienced either true relapse as evidenced by worsening radiographs (n = 9) or disseminated infection (n = 4). About half of those with rebounded titers also had recurrence of symptoms, which prompted retreatment in three-quarters of patients. In addition, one-half of patients without symptoms also received additional antifungal therapy, begun simply because the titer had increased by 2 or more dilutions. Whether antifungal therapy is indicated in those with asymptomatic serologic rebound is unclear, and experts within the endemic area may disagree on this issue in the absence of data. In patients who do not have other objective evidence of relapsed infection, such as new or worsening radiographic abnormalities, our experience is that it may be reasonable to closely monitor such patients without additional treatment. If an observational stance is taken, we suggest clinical reevaluation every few months for 1–2 (or more) years, with the duration and frequency depending on the patient’s clinical well-being, their understanding of what symptoms should prompt provider contact, and the height of the CF titer.

Four patients with serologic rebound developed extra-thoracic dissemination and three experienced symptomatic relapses associated with the identification of coccidioidal meningitis within 5 months of discontinuation of the initial course of antifungal treatment. These results suggest that any patient who develops a headache, nausea, or any neurological symptoms in association with an increasing complement fixation titer after antifungal discontinuation for primary pulmonary coccidioidomycosis should be evaluated for central nervous system disease, including cerebrospinal fluid examination via lumbar punctures and brain and spinal cord imaging [8].

Because the concepts in complement fixation serology were observed and articulated prior to the development and routine use of azole antifungal treatment, recent observations have refined our understanding of complement fixation titers in the antifungal era. Thompson et al. demonstrated that the initiation of antifungal treatment within 2 weeks of symptom onset was associated with a higher likelihood to an abrogated IgG response, making the serologic diagnosis of coccidioidomycosis more difficult [9]. Separately, a study was conducted of 438 patients who were treated for a spectrum of coccidioidal illnesses, among whom 248 had uncomplicated pulmonary coccidioidomycosis. Among the 248, 9% experienced at least a 2-dilution serologic recurrence during the follow-up period. [4]. This serologically relapsed group was not otherwise described with respect to symptoms or sequelae of coccidioidal infection, if any, and what treatment may have occurred because of the serologic recurrence [4]; however, the phenomenon of rebounded complement fixation serology following antifungal treatment appeared at a similar frequency as the current study.

The rising titer as a single marker, without other objective evidence of recurrent infection, may be a phenomenon unrelated to reactivated disease. The mechanism of serologic rebound is not yet articulated with certainty, but numerous hypotheses have been described. Galgiani postulated several possible mechanisms for a rise in serologic results following treatment, including endemic re-exposure to the organism, temporary loss of host control resulting from a transient local immunosuppression, or subclinical granuloma degradation resulting in vivo re-exposure [10]. Thompson and colleagues observed that fluconazole therapy may suppress the development of a coccidioidal IgG response, leading to the hypothesis that asymptomatic serologic rebound in at least some cases is the result of the loss of antibody suppression. [9] Similarly, we postulate that the withdrawal of the fungistatic triazole treatment potentially offers a new opportunity for fungal growth, which then further stimulates an immune response. Finally, of course, the rise in complement fixation titer following discontinuation of treatment may be an early sign of relapsed coccidioidal infection due to failure of immunological control [10].

Our study has notable limitations. It was retrospective and conducted in a tertiary care institution, of which the population is predominately white, and included patients under the care of multiple practitioners without standardized testing or treatment protocols. We acknowledge that the differences in treatment versus non-treatment of asymptomatic patients with rebounded titers (in the absence of objective data that support reactivated infection) complicates the interpretation of our results. We limited our study to non-immunosuppressed and non-pregnant adult patients, and the results may not be generalizable to other populations. Additionally, our search was limited to selected diagnosis codes, and we may have missed other cases that were coded differently. During the study timeframe, our reference lab made changes in the conduct of the complement fixation process that may have affected our results.

We conclude from this study that otherwise healthy patients who receive a discrete course of antifungal treatment for uncomplicated pulmonary coccidioidomycosis and then experience rebounded serology after stopping antifungal treatment, should be monitored over time. The rising titer in itself, without objective evidence of recurrent infection, may be a phenomenon unrelated to reactivated disease. Many patients will not require antifungal treatment, especially if asymptomatic, but all should be aware of symptoms that could signal recurrence or dissemination. Although more likely to have a longer follow-up for coccidioidomycosis, most will not go on to have complicated endpoints.

## Figures and Tables

**Table 1:** Comparison of 58 patients with treated primary pulmonary coccidioidomycosis, whose serology rebounded following treatment, compared with matched controls.

Characteristic	Patients with Serologic Rebound (%) n = 58	Patients without Serologic Rebound Controls (%) n = 58	*p*-Value *
Age in years, median, (range)	63.5 (28–86)	61 (22–80)	0.61 ^ɣ^
Male Sex	40 (70)	37 (63.8)	0.7
Race			
White	43 (74)	43 (74)	1.0
Asian	5 (8.6)	5 (8.6)	1.0
Filipino	5 (8.6)	5 (8.6)	1.0
African American	3 (5.2)	3 (5.2)	1.0
Native American	1 (1.7)	1 (1.7)	1.0
Other	1 (1.7)	1 (1.7)	1.0
Hispanic Ethnicity	4 (6.9)	4 (6.9)	1.0
Duration follow-up, months	29.5 (11–139)	25.0 (1–111)	0.027 ^ɣ^
Initial coccidioidal symptoms			
Cough	48 (82.7)	39 (67.2)	0.08
Fever	42 (72.4)	39 (67.2)	0.7
Shortness of Breath	31 (53.4)	23 (39.6)	0.2
Night sweats	24 (41.3)	20 (34.4)	0.6
Rash	20 (34.4)	11 (18.6)	0.09
Headache	16 (27.5)	12 (20.6)	0.5
Chest pain	10 (17.4)	9 (15.5)	1.0
Initial CF titer (range)	1:16 (1:2–1:256) ^£^	1:8 (1:2–1:128) ^§^	0.18
Initial treatment duration, median, in months (range)	6.4 (1–33)	6.0 (1.5–47)	0.28 ^ɣ^
Coccidioidal symptoms after 1st course of treatment	30 (51.7)	13 (22.4)	0.0001
≥2 courses of antifungal treatment	37 (63.7)	5 (8.6)	0.0001
Dissemination	4 (6.8)	1 (1.7)	0.4
Meninges	3 (5.1)	1 (1.7)	0.6
Skeletal	1 (1.7)	0	1.0

* *p*-value calculated using the Fisher exact test, unless otherwise noted; ^ɣ^ *p*-value calculated using *t*-test; ^£^ n = 54; ^§^ n = 57.

## Data Availability

The data presented in this study can be requested from the corresponding author. The data are not publicly available due to privacy issues.

## Supplementary Materials

The following supporting information can be downloaded at: https://www.mdpi.com/article/10.3390/jof9090901/s1, Table S1: Characteristics of 58 patients with serologic rebound after stopping antifungal treatment for primary pulmonary coccidioidomycosis; Table S2: Comparison of characteristics in patient whose rebounded serology was manifested by disseminated infection.
