# Supplementary material for: Serologic Rebound after Stopping Azoles for Primary Pulmonary Coccidioidomycosis: A Case-Controlled Observational Study
Source: J Fungi (Basel). 2023 Sep 1;9(9):901. doi: 10.3390/jof9090901 (PMC10532748; doi:10.3390/jof9090901)
Supplement: Supplementary file 1 [file jof-09-00901-s001.zip › jof-2541492-supplementary.pdf]

Supplement

Table S1. Characteristics of 58 patients with serologic rebound after stopping antifungal treatment for primary pulmonary coccidioidomycosis.

Characteristic	Asymptomatic Rebound, Number (%) n=28**	Symptomatic Rebound (i.e., Relapse), Number (%) n=30**	Total Number, (%) n=58	P value*
Age in years, median, (range)	66 (28-80)	62 (28-86)	63.5 (28-86)	0.91
Male Sex	20 (71.4)	20 (67)	40 (69)	0.8
Race				
White	17 (60.7)	26 (86.6)	43 (74)	0 .03
Asians	4 (14.2)	1 (3.3)	5 (8.6)	0.2
Filipino Asian	2 (7.1)	4 (13.3)	6 (10.3)	0.7
African American	3 (10.7)	0 (0.0)	3 (5.2)	0.1
Native American	1 (3.6)	0 (0.0)	1 (1.7)	0.5
Other	1 (3.6)	0 (0.0)	1 (1.7)	0.5
Hispanic Ethnicity	3 (10.7)	1 (3.3)	4 (6.9)	0.3
Past Medical History				
Remote Solid Cancer	5 (17.9)	2 (6.7)	7 (12.5)	0.4
Remote Hematological malignancy	2 (7.1)	1 (3.3)	3 (5.4)	1.0
Diabetes	9 (32.1)	5 (16.7)	14 (25)	0.3
Short term Steroid given at Initial PPC illness	8 (28.5)	4 (13.3)	12 (20.6)	0.2
Active Smoker	2 (7.1)	2 (6.7)	4 (6.9)	1.0
Never Smoked	19 (67.9)	13 (43.3)	32 (55.1)	0.07
Initial PPC Symptoms				
Cough	26 (92.8)	22 (73.3)	48 (82.7)	0.08
Fever	20 (71.4)	22 (73.3)	42 (72.4)	1.0
Shortness of Breath	16 (57.1)	15 (50)	31 (53.4)	0.6
Night sweats	12 (42.8)	12 (40)	24 (41.3)	1.0
Rash	8 (28.6)	12 (40)	20 (34.4)	0.4
Headache	7 (25)	9 (30)	16 (27.5)	0.8
Chest pain	4 (14.2)	6 (20)	10 (17.4)	0.7
Initial radiology				
Nodules	10 (35.7)	20 (66.8)	30 (51.7)	0.5
Infiltrates	18 (64.3)	13 (43.3)	31 (53.4)	0.1
Cavity	2 (7.1)	3 (10)	5 (8.6)	1.0
Mass	4 (14.2)	6 (20)	10 (17.2)	0.7
Lymphadenopathy	12 (42.8)	11 (36.6)	23 (39.6)	0.8
Initial CF titer, median, (range)	24.0 (2-256) (n=27)	16.0 (2-128) (n=27)	16 (2-256)	0.16
Months to develop rebound serology post discontinuation of initial antifungal treatment, median (range)	4.0 (0.6-21.6)	3.0 (0.6-18.2)	3.5 (0.6-21.6)	0.66
Rebound CF titer, median, (range)	24.0 (4 – 128)	16.0 (4-256)	16.0 (4-256)	.98

Treatment reinitiated at re-bounded serology	14 (50)	23 (76.7)	37 (63.7)	0.05
Post rebound End point				
Dissemination	1 (3.6)	3 (10.7)	4 (7.1)	0.6
Meninges	0 (0.0)	3 (10.7)	3 (5.4)	0.2
Skeletal	1 (3.6)	0 (0.0)	1 (1.8)	1.0
Resolved infection	7 (25)	8 (28.5)	15 (26.8)	1.0
Total Follow-up duration (In years)	Median: 2.4 (1.3 – 10.9)	Median: 2.7 (1.0 – 11.6)	Median: 2.5 (1.0 – 11.6)	0.9

*Calculated using Fisher's exact test for categorical variables and t-test for continuous variables.
**Unless otherwise specified.
PPC, primary pulmonary coccidioidomycosis.

Table S2. Comparison of characteristics in patient whose rebounded serology was manifested by disseminated infection.

Characteristic	Disseminated number (%) n=4	Non disseminated Number (%) (n=54)	Total Number (%) n=58	P value*
Age (in years)	Median: 66 (59-73)	Median: 63.5 (28-86)	Median: 70 (28-86)	0.44
Male Sex	4 (100)	36 (66.7)	40 (68.9)	0.3
Race				
White	4 (100)	39 (72.2)	43 (74.1)	0 .5
Asians	0 (0)	5 (9.3)	5 (8.6)	1.0
Filipino Asian	0 (0)	6 (11.1)	6 (10.3)	1.0
African American	0 (0)	3 (5.7)	3 (5.2)	1.0
Native American	0 (0)	1 (2)	1 (1.7)	1.0
Other	0 (0)	1 (1.8)	1 (1.7)	1.0
Hispanic Ethnicity	0 (0)	4 (7.4)	4 (6.9)	1.0
Past Medical History				
Remote Solid Cancer	1 (25)	6 (11.1)	7 (12.1)	0.4
Remote Hematological malignancy	0 (0)	3 (5.6)	3 (5.2)	1.0
Diabetes	0 (0)	14 (25.9)	14 (24.1)	0.5
Short term Steroid given at Initial PPC illness	0 (0)	12 (22.2)	12 (20.7)	0.5
Active Smoker	1 (25)	4 (7.4)	5 (8.6)	0.3
Never Smoked	2 (50)	29 (53.7)	32 (55.2)	1.0
Initial PPC Symptoms				
Cough	2 (50)	46 (85.1)	48 (82.8)	0.1
Fever	2 (50)	40 (74.0)	40 (69.0)	0.3
Shortness of Breath	2 (50)	29 (53.7)	31 (53.4)	1.0
Night sweats	2 (50)	22 (40.7)	24 (41.3)	1.0
Rash	1 (25)	19 (35.2)	20 (34.5)	1.0
Headache	0 (0)	16 (29.6)	16 (27.6)	0.6
Chest pain	0 (0)	10 (18.5)	10 (17.2)	1.0
Initial radiology				
Nodules	4 (100)	26 (48.1)	30 (51.7)	0.1
Infiltrates	0 (0)	31 (57.4)	31 (53.4)	0.04
Cavity	0 (0)	5 (9.2)	5 (8.6)	1.0
Mass	1 (25)	9 (16.6)	10 (17.2)	0.5
Lymphadenopathy	1 (25)	22 (40.7)	23 (39.6)	1.0
Initial CF titer	Median: 12 (8-16) (n=3)	Median: 16 (2-256) (n=49)	Median: 16 (2-256)	0.5
Time to develop Rebound serology post discontinuation of initial antifungal treatment (in months)	Median: 1.7 (0.7-2.1)	Median: 4.2 (0.6-21.6)	Median: 3.5 (0.6-21.6)	0.08
Rebound CF titer	Median: 40 (16 – 256)	Median: 16 (4-128)	Median: 16 (4-256)	0.005
Treatment reinitiated at Rebound Serology	4 (100)	33 (61.1)	37 (63.7)	0.2
Post rebound End point				
Resolved	0 (0)	17 (28.8)	17 (29.3)	0.3

Continued follow up	4 (100)	31 (57.4)	31 (53.4)	0.14
Lost to follow up	0 (0)	4 (7.6)	4 (6.8)	1.0
Continued treatment	4 (100)	2 (3.8)	6 (10.3)	0.0006
Total Follow-up duration (In years)	Median: 2.8 (1.2 – 10.9)	Median: 2.5 (1.0 – 11.6)	Median: 2.5 (1.0 – 11.6)	0.7

*Calculated using Fisher's exact test for categorical variables and t-test for continuous variables.
PPC, primary pulmonary coccidioidomycosis.
